# Correlation between the corrosion rate and electrochemical noise energy of copper in chloride electrolyte

**DOI:** 10.1039/c8ra01203b

**Published:** 2018-05-24

**Authors:** Chenxi Yi, Xiaoqing Du, Yumeng Yang, Benfeng Zhu, Zhao Zhang

**Affiliations:** Department of Chemistry, Zhejiang University Hangzhou Zhejiang 310027 China yichenxi2013@163.com

## Abstract

An electrochemical noise technique has been applied to describe the corrosion process of copper. The results show that the sampling frequency clearly changes both the energy distribution plot and the power spectral density spectra, which should be taken into consideration strictly and logically before an electrochemical noise test. The corrosion energy, (*E*_c_), deduced using the fast wavelet transform method showed a similar variation trend with corrosion rate. Hence, the proposed parameter *E*_c_ represents the corrosion rate or severity.

## Abstract 2

Sampling frequency clearly changes EDP and PSD spectra and EN corrosion energy shows a similar variation trend with corrosion rate.

## Graphical abstract

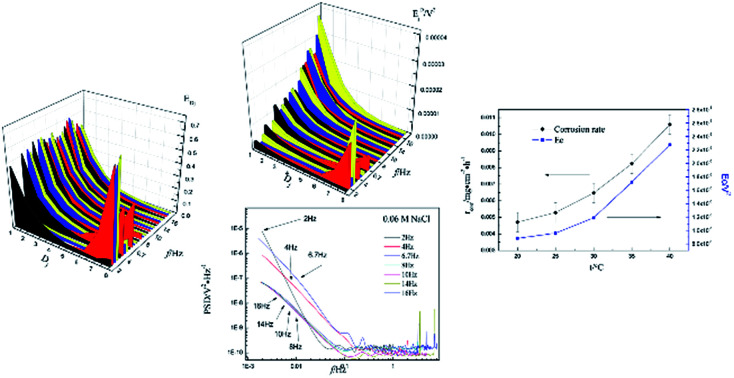

## Introduction

Electrochemical noise (EN) is the generic term given to the fluctuation of current and potential, and corrosion engineering is regarded as one of its important fields of application.^1–6^ Fluctuations of electrode potential are connected to local anodic and cathodic reactions and are a consequence of events occurring at flaws (such as pitting nucleation, propagation, and re-passivation), which are strictly related to the corrosion process.^7^

From a physicochemical viewpoint, the electrode potential is defined as the change in Gibbs energy when a charged particle transfers from the infinite into an electrode, including both the electrical and chemical work done during the process, whereas, potential only consists of the electrical work in the above transfer process. Therefore, the variation in the electrode potential definitely comes from the energy exchange between the electrode system and the environment.^8^ Additionally, the energy is divided into potential energy and kinetic energy, and both of them can be converted into each other. Potential energy is “inert” and only reflects the stability of objects, meanwhile kinetic energy is “active” and directly depends on velocity. Similarly,^9–11^ the fluctuation of electrode potential always simultaneously consists of “slow DC drift” and a “fast random non-equilibrium fluctuation signal”. The former is the traditional electrode potential, which indicates the thermodynamic stability; whilst the latter is designated as electrochemical noise, which represents the speed of the electrode reaction.

EN data are usually analyzed using FFT (fast Fourier transform) and MEM (maximum entropy method) techniques to obtain PSD (power spectral density) plots,^3,12^ or using the FWT (fast wavelet transform) technique to obtain an EDP (energy distribution plot, *i.e.*, the plot of the relative energy accumulated by each crystal *vs.* the crystal name) or an RP-EDP (re-plotted EDP by discounting the energy contribution of the smooth coefficient set from the ensemble signal energy).^1^ Three parameters can be obtained from PSD plots: the slope of the high frequency linear region (*k*), the critical frequency or the cut-off frequency (*f*_c_) and the low frequency plateau (*W*).^13^ Generally,^3^*k*, *f*_c_ and *W* of potential PSD are related to the severity of the corrosion to some extent. *k* is regarded as a source of mechanistic information and is used to differentiate between general and localized corrosion.^13–15^ For EDP plots, the interval range (or scale range) of each crystal (*j*) is given by,^2,16^

1 (*C*_1_^*j*^, *C*_2_^*j*^) = (2^*j*^Δ*t*, 2^*j*−1^Δ*t*)

The mainstream EN practices^17–20^ establish the relationship between the EDP and corrosion morphology. However, the scale range is dependent on Δ*t* (Δ*t* = 1/*f*) and the features of the EDP may vary with different Δ*t* even for the same corrosion morphology. Therefore, when obtaining an EDP, using a selection of adequate sampling frequencies (*f*) is logical.

While investigating the corrosion behaviour of mild steels in saturated calcium hydroxide with 20 g L^−1^ CaCl_2_, Searson and Dawson^15^ found that there existed a relationship between the corrosion rate (*r*_corr_) (obtained from weight loss) and the standard deviation of the potential noise with a correlation of 10^−5^, *i.e.*, standard deviation × 10^−5^ = *r*_*corr*_ (mpy). Although the comparison of short-term EN (1024 s in their work) with long-term weight loss (an average of several days) seems inappropriate, their pioneering work undoubtedly demonstrates a correlation between EN features and the corrosion rate.

Encouraged by Searson and Dawson,^15^ and based on previous reports^21,22^ that pitting definitely occurred when copper was immersed into chloride solutions, this study was devoted to finding the influence of the adopted EN sampling frequency on the characteristics of both EDP and PSD plots, which are obtained from MEM and FWT analyses of the same electrochemical potential noise, and especially to probing the correlation between the corrosion rate and the electrochemical noise energy.

## Theory of EN analysis

EN can be analysed using FFT, MEM and FWT techniques. When compared with FFT or MEM techniques, FWT can remove the DC trend without any observed signal distortion.^23^ Therefore, the conclusions obtained using FWT are more rational. Moreover, the EN energy (*E*) of each wavelet crystal of a different time-scale can be obtained using the FWT technique.^18,24,25^ After removing the energy contribution of DC drift, the mainstream RP-EDP plot could be obtained from *E*_*j*_^*D*^.^26^2
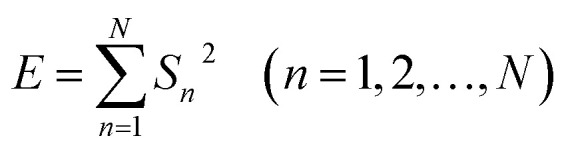
3
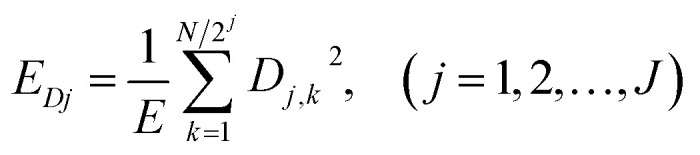
4
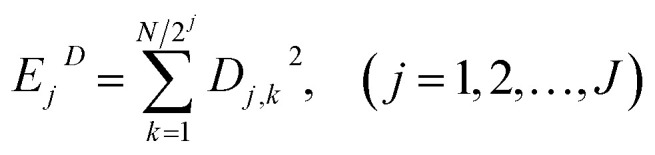
where *E*_*j*_^*D*^ is the energy value of the *D*_*j*_ crystal with units of V,^2^ and *E*_*Dj*_ is the value after unitization. Generally, the *E*_*j*_^*D*^ of a specific timescale (or frequency range) can be related to a certain corrosion process.^26,27^ For example, the metastable pitting/nucleation process always takes place prior to the nuclei growth process, both of which are much faster than the diffusion process, which therefore results in the large *E*_*j*_^*D*^ of low *j* values.^2,19,20,28^ It should be noted that the timescale range of *D*_*j*_ is strictly dependent on Δ*t* (or frequency) (eqn (1)), therefore each *D*_*j*_ partition of the RP-EDP plot will change along with the variation of *f*.

## Experimental

Corrosive electrolytes (NaCl at different concentrations, 0.03 M, 0.06 M, 0.09 M and 0.12 M, and 0.06 M HCl) were prepared from analytical grade NaCl or HCl and double distilled water. The working electrode (pure copper) was mechanically cut and embedded into Teflon, leaving an exposed area of 0.5 cm^2^ as the working surface. Prior to each experiment, the working surface was successively abraded using sand paper from 400 to 1200 grit, polished to a mirror with 2.5 μm diamond paste, rinsed with distilled water in an ultrasonic cleaner for about 3 min (KQ5200B, Youyi Instrument Co., Ltd., China), degreased with acetone and finally dried under a cool N_2_ flow.

EN was monitored as a function of time between the working electrode and the reference electrode (SCE) for the 1^st^ hour, using the Powerlab/4sp (made in Australia) electrochemical interface through a GP amplifier controlled by Chart 5 software for the Windows XP operating system. This instrument is equipped with analog/hardware filters including an AA filter (anti-aliasing low-pass filters) to remove high frequency components before the signal is digitized, so that the acquisition of false data can be avoided.^11^ The EN records were collected at different sampling intervals (sampling frequency, *f*).

The weight loss of copper was measured according to the standard ISO 8407:2009, IDT. Copper coupons with dimensions of 100 mm × 80 mm × 0.2 mm were sectioned, and a water bath was used to maintain a specific temperature. SEM (SIRION, FEI Company, made in Holland) was utilized to examine the corrosion morphologies of the specimens.

## Results and discussion

### Influence of EN testing frequency

**Fig. 1:**
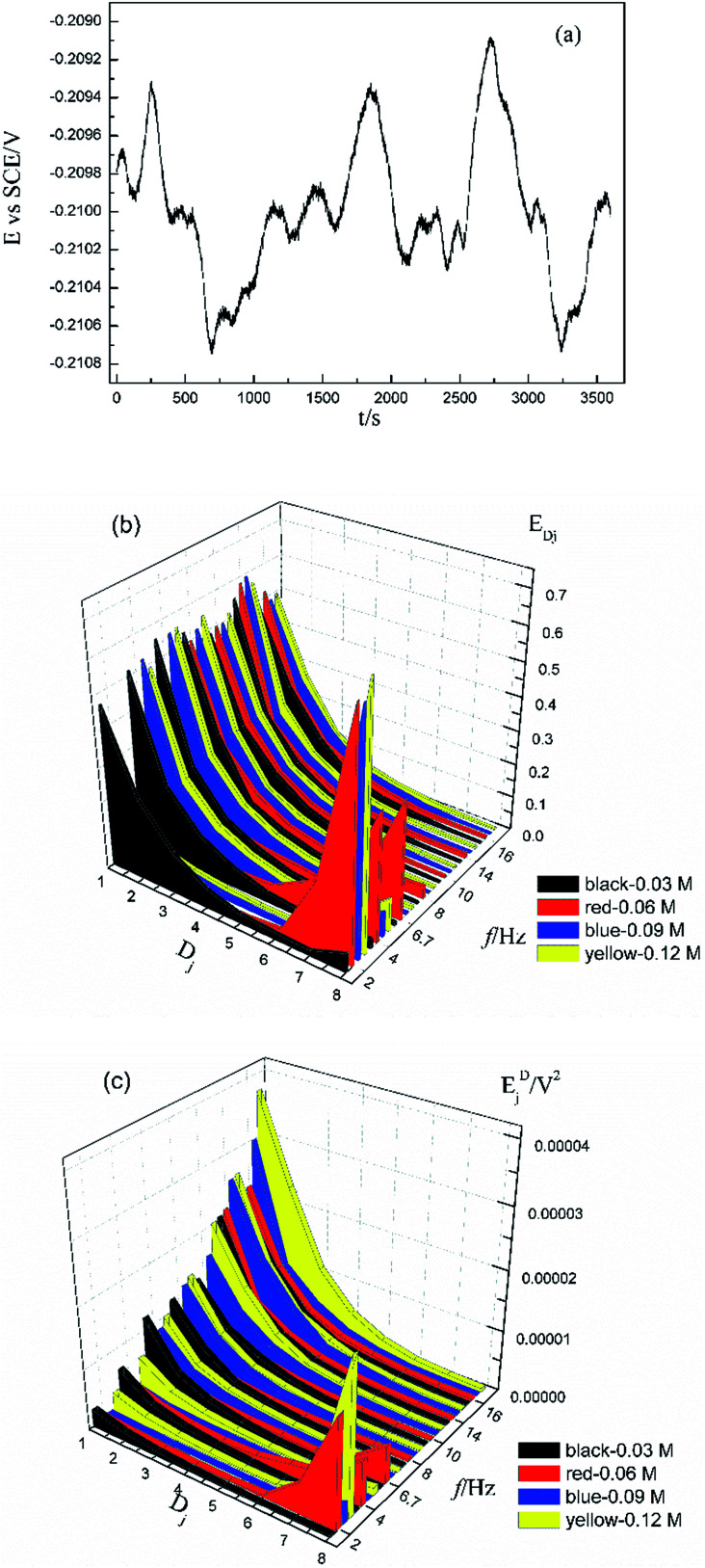
(a) Typical *E*–*t* curve at a frequency of 2 Hz, (b) *E*_*Dj*_ map (with unitization) and (c) *E*_*j*_^*D*^ map with varied sampling frequencies and *C*_NaCl_: black–0.03 M, red–0.06 M, blue–0.09 M and yellow–0.12 M.

Fig. 1a shows the typical time-domain potential noise of the 1^st^ hour for Cu corroding in 0.06 M NaCl neutral solutions at 20 °C, acquired at a commonly used data-sampling frequency (*f*) of 2 Hz. Fig. 1b and c show the RP-EDP plots of the noise generated during the same corrosion process at various concentrations of NaCl with different *f* (*T* = 20 °C). The difference is that the data shown in Fig. 1b were obtained after unitization (eqn (3)) whereas the data shown in Fig. 1c were obtained without unitization (eqn (4)). For 0.06 M, 0.09 M and 0.12 M NaCl (in Fig. 1b), the RP-EDPs remain the same and the maximum (relative) energy is in *D*_1_–*D*_3_ when *f* ≥ 8 Hz while it is in *D*_7_–*D*_8_ when *f* < 4 Hz. In 0.03 M NaCl, the maximum energy is in *D*_1_–*D*_3_ at each testing frequency.

**Fig. 2:**
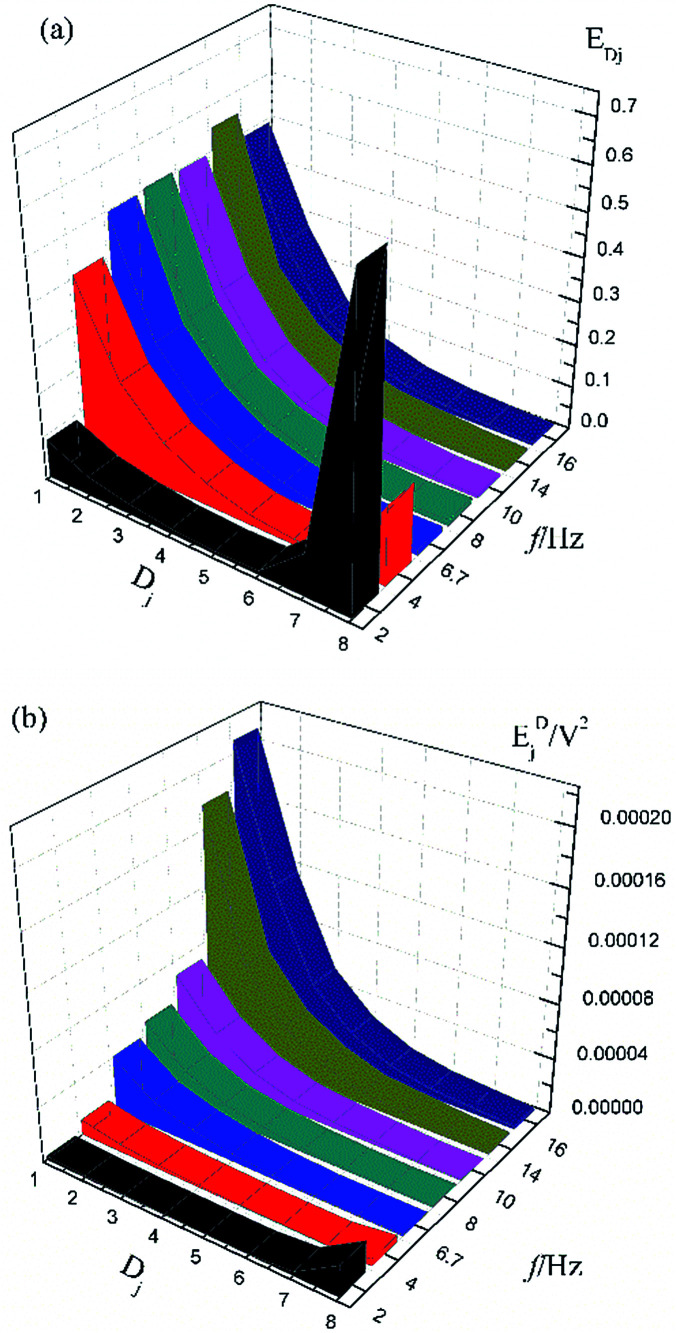
(a) *E*_*Dj*_ map (with unitization) and (b) *E*_*j*_^*D*^ map at different sampling frequencies in 0.06 M HCl solution.

Fig. 2 shows the RP-EDP plots with and without unitization for Cu corroding in 0.06 M HCl solutions. The maximum energy of RP-EDP is in *D*_7_–*D*_8_ when *f* < 4 Hz, while it is in *D*_1_–*D*_3_ when *f* ≥ 4 Hz. Considering Fig. 1 and 2, the characteristics of the RP-EDP plot are significantly influenced by sampling frequency. With increasing *f*, the maximum relative energy of the RP-EDP is in *D*_1_–*D*_3_ regardless of the corrosion ionic strengths.

**Fig. 3:**
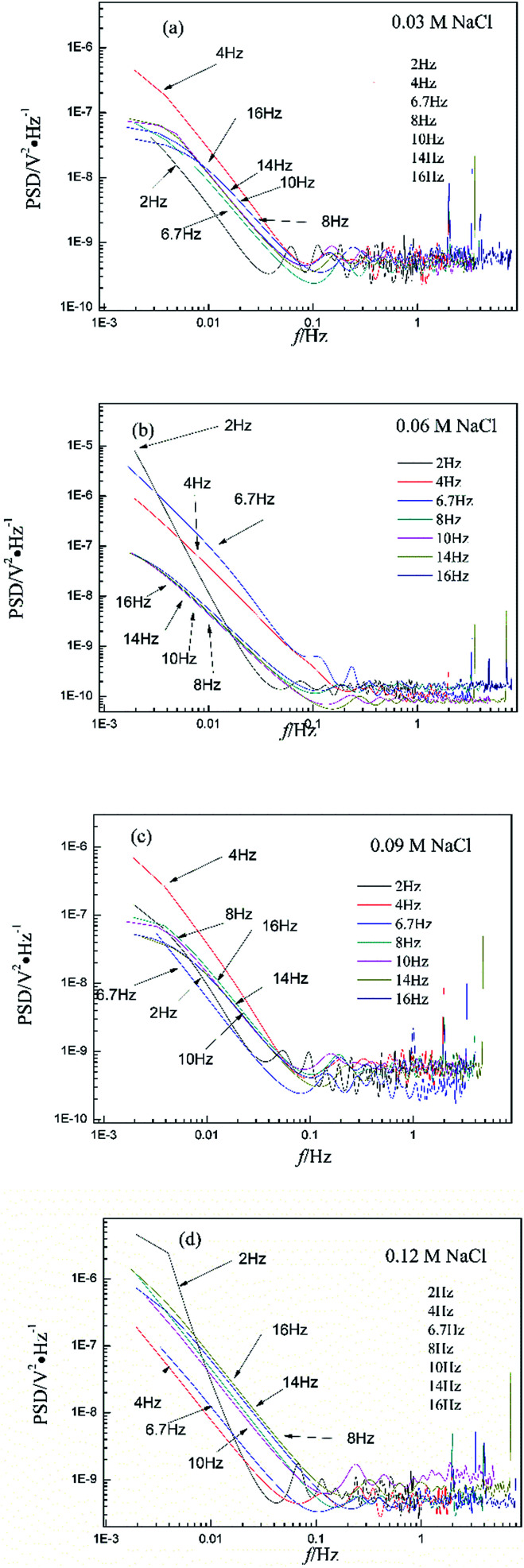
Influence of frequency effects on the PSD spectra in different concentrations of NaCl.

**Fig. 4:**
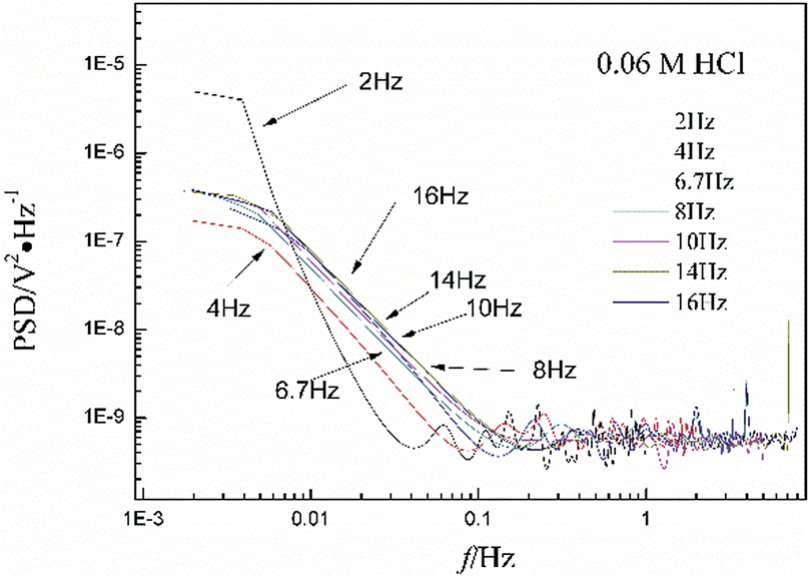
Influence of frequency effects on the PSD spectra in 0.06 M HCl.

Fig. 3 shows the PSD analysed using the MEM technique in different concentrations of NaCl and Fig. 4 shows the PSD in 0.06 M HCl. It is evident that PSD parameters (*f*_c_, *W*, and *k*) are also dependent on the adopted EN sampling frequency. In 0.06 M, 0.09 M and 0.12 M NaCl, these PSD parameters retain approximately equal values while *f* ≥ 8 Hz, whereas others (<8 Hz) are distinct from each other. Meanwhile the turning frequency point is 6.7 Hz in 0.03 M NaCl and 0.06 M HCl. These results are in accordance with the RP-EDP plots (Fig. 1 and 2), and also demonstrate the significant influence of the sampling frequency on the EN results.

From a physicochemical viewpoint, the corrosion process should mainly depend on the natures of both the material (such as resistivity) and the environment (such as ambient temperature and erosive particles), but independent of the testing tools (such as the adopted EN sampling frequency). Additionally, the optimal or appropriate EN sampling frequency may be related to the investigated materials and their environment, and also possibly the research target of the researchers. Considering Fig. 1–4, the optimal testing frequency should be 6.7 Hz in 0.03 M NaCl and 0.06 M HCl and 8 Hz in 0.06 M, 0.09 M and 0.12 M NaCl. Previous reports^14,15,29,30^ claimed that an EN sampling frequency of 1–4 Hz (Δ*t* = 0.25–1 s) seems to be adequate, a reason for which may be that their efforts focused on the noise resistance deduced from PSD spectra of potential and current noise.

### Relationship between EN features and corrosion rate

**Fig. 5:**
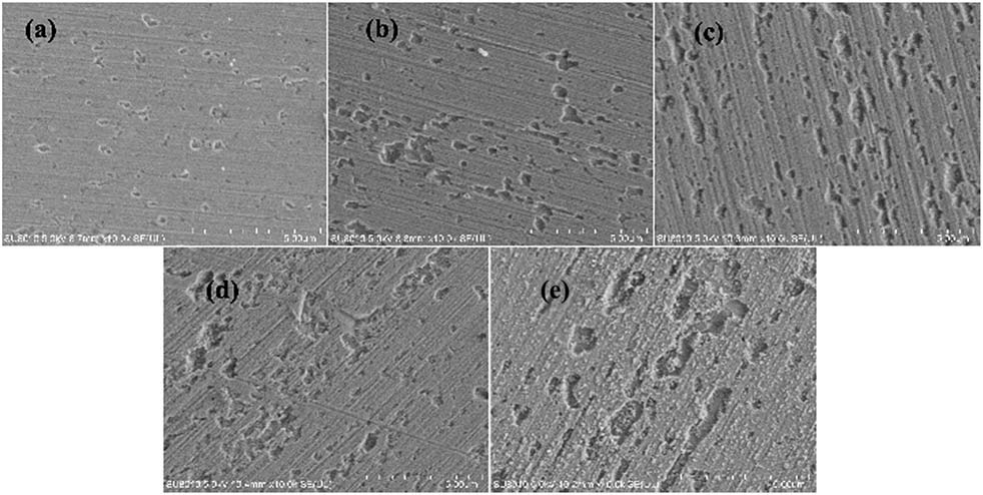
SEM images of the Cu morphologies after corroding for 1 h in 0.06 M NaCl solutions at different temperatures: (a) 20 °C, (b) 25 °C, (c) 30 °C, (d) 35 °C and (e) 40 °C.

**Fig. 6:**
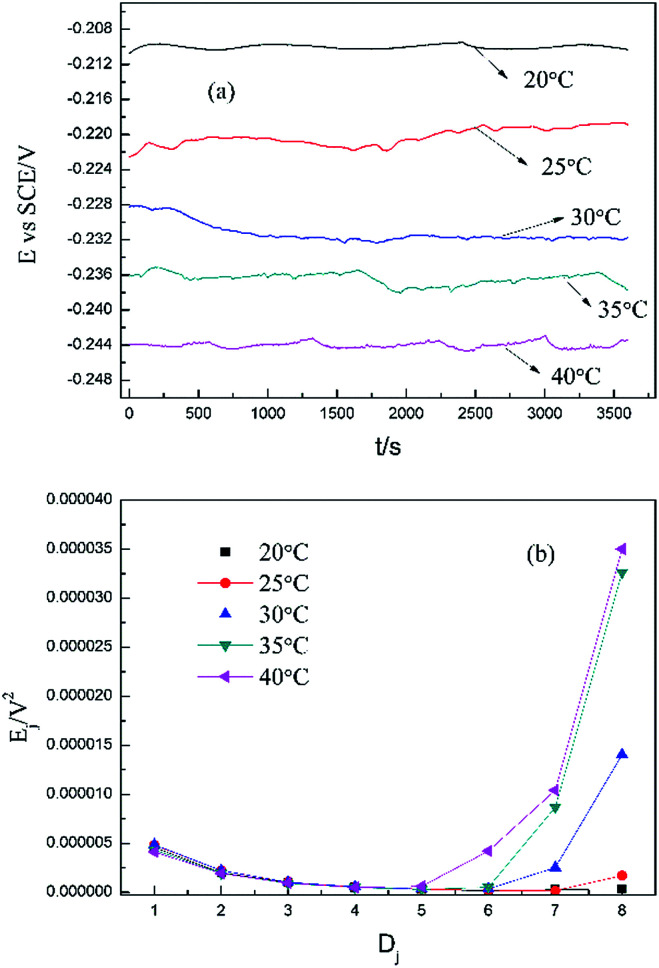
(a) Potential noise and (b) corresponding RP-EDP plots for copper corroding in 0.06 M NaCl at different temperatures (1^st^ h, *f* = 8 Hz).

Based on the above analyses and inspired by Searson and Dawson,^15^ the corroded morphologies (Fig. 5), the weight loss and the potential noise (Fig. 6a) of Cu corroding in 0.06 M NaCl neutral solutions at different temperatures were acquired, and the RP-EDPs without unitization were obtained (Fig. 6b). When performing EN tests, *f* = 8 Hz was adopted according to previous sections. Additionally, in order to ensure the comparability between the weight loss and the EN features, the same initial 1 h corroding period was selected as the timescale for both.

**Fig. 7:**
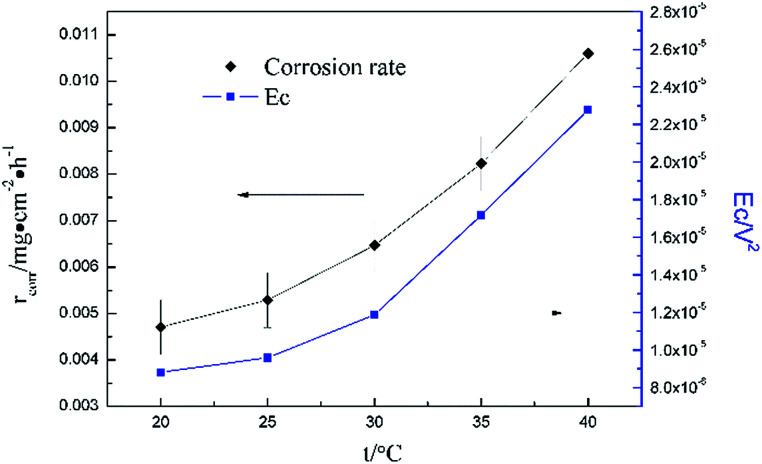
Dependence of corrosion energy *E*_c_ and *r*_corr_ on temperature in 0.06 M NaCl.

Fig. 5 clearly shows the pitting corrosion type, and the diameter and depth of the pits increase with temperature, which intuitively represents an enhancement of the corrosion rate. Since the passivation time of a metastable pit is typically no longer than 20 s,^31^ and *D*_8_ mainly reflects the information of diffusion at *f* = 8 Hz,^32^ the sum of the energy (Fig. 6b) deposited in crystals *D*_1_ ∼ *D*_7_ (*E*_c_), which represents the active pitting energy and therefore should reflect the severity of the corrosion of copper,^8,33^ is calculated and plotted *versus* the temperature (Fig. 7).

5 *E*_c_ = *E*_1_^*D*^ + *E*_2_^*D*^ + *E*_3_^*D*^ + *E*_4_^*D*^ + *E*_5_^*D*^ + *E*_6_^*D*^ + *E*_7_^*D*^

*r*_corr_ (units mg cm^−2^ h^−1^) obtained from the weight loss is also repeatedly plotted in Fig. 7, and both *r*_corr_ and *E*_c_ increase with the elevation of the temperature. In other words, a good parallel relationship between the *r*_corr_ and *E*_c_ can be confirmed during the investigated timescale.

## Conclusions

The electrochemical noise technique has been applied to describe the corrosion process of copper. The results show that the sampling frequency clearly changes both the EDP and the PSD spectra, before establishing the relation between the EDP and surface morphology, or the PSD spectra and corrosion kinetics, the optimal testing frequency should be taken into consideration strictly and logically.

The corrosion energy, *E*_c_, deduced from the FWT method, shows a similar variation trend with the corrosion rate. Hence, electrochemical noise offers a nondestructive on-line monitoring process, which can be easily carried out, and the proposed parameter *E*_c_ represents the corrosion rate or severity.

## Conflicts of interest

There are no conflicts to declare.

## Supplementary Material
